# Diversity of the free-living marine and freshwater Copepoda (Crustacea) in Costa Rica: a review

**DOI:** 10.3897/zookeys.457.6820

**Published:** 2014-11-25

**Authors:** Álvaro Morales-Ramírez, Eduardo Suárez-Morales, Marco Corrales-Ugalde, Octavio Esquivel Garrote

**Affiliations:** 1 Centro de Investigación en Ciencias del Mar y Limnología (CIMAR); 2 Escuela de Biología, Universidad de Costa Rica, 11501-2060 San José, Costa Rica; 3 Colegio de la Frontera Sur (ECOSUR), Chetumal, Mexico; 4 Licenciatura en Biología, Escuela de Biología, Universidad de Costa Rica, 11501-2060 San José, Costa Rica

**Keywords:** New records, biodiversity, Pacific, Caribbean, microcrustaceans, biogeography

## Abstract

The studies on marine copepods of Costa Rica started in the 1990’s and focused on the largest coastal-estuarine systems in the country, particularly along the Pacific coast. Diversity is widely variable among these systems: 40 species have been recorded in the Culebra Bay influenced by upwelling, northern Pacific coast, only 12 in the Gulf of Nicoya estuarine system, and 38 in Golfo Dulce, an anoxic basin in the southern Pacific coast of the country. Freshwater environments of Costa Rica are known to harbor a moderate diversity of continental copepods (25 species), which includes 6 calanoids, 17 cyclopoids and only two harpacticoids. Of the +100 freshwater species recorded in Central America, six are known only from Costa Rica, and one appears to be endemic to this country. The freshwater copepod fauna of Costa Rica is clearly the best known in Central America. Overall, six of the 10 orders of Copepoda are reported from Costa Rica. A previous summary by 2001 of the free-living copepod diversity in the country included 80 marine species (67 pelagic, 13 benthic). By 2009, the number of marine species increased to 209: 164 from the Pacific (49% of the copepod fauna from the Eastern Tropical Pacific) and 45 from the Caribbean coast (8% of species known from the Caribbean Basin). Both the Caribbean and Pacific species lists are growing. Additional collections of copepods at Cocos Island, an oceanic island 530 km away of the Pacific coast, have revealed many new records, including five new marine species from Costa Rica. Currently, the known diversity of marine copepods of Costa Rica is still in development and represents up to 52.6% of the total marine microcrustaceans recorded in the country. Future sampling and taxonomic efforts in the marine habitats should emphasize oceanic environments including deep waters but also littoral communities. Several Costa Rican records of freshwater copepods are likely to represent undescribed species. Also, the biogeographic relevance of the inland copepod fauna of Costa Rica requires more detailed surveys.

## Introduction

Copepods are a highly diverse group of crustaceans; over 13 000 species of this subclass have been described (Boxshall and Halsey 2004, Boxshall and Defaye 2008) and many more are added each year. Copepods are also one of the most abundant metazoans in the world. Up to 60–80% of the marine zooplankton biomass in neritic and oceanic environments is represented by copepods (Casanova et al. 1982). This abundance is related to the success of the planktonic forms in colonizing the oceanic water column (Boxshall and Halsey 2004). Huys and Boxshall (1991) summarized the economic and biological importance of copepods.

The free-living forms inhabit a wide variety of aquatic environments including also those with extreme conditions of salinity and temperature. They are common members of the biotas of subterranean waters (Pipan 2005), anchialine systems (Suárez-Morales and Iliffe 2005), hypersaline waters (Brucet et al. 2009), and bromeliads, among others (Reid 1986).

Although the importance of the free-living copepods is clear, information on their diversity and distribution is scarce and scattered in many regions. In the case of marine forms, Mauchline (1998) listed 13 Large Marine Ecosystems (LME) that have some data regarding their copepod fauna, but information on most of the remaining 75% of LMEs remains largely incomplete. Similarly, the freshwater environments harbor a diverse copepod fauna; in general, the knowledge of the group in the Americas is asymmetrical, Central America being the least studied subregion in the continent. As part of the Neotropical region with a high continental copepod diversity (Boxshall and Defaye 2008), studies of the group in Costa Rica have been intermittent (Collado et al. 1984a, b, Suárez-Morales and Reid, unpubl. data), but new efforts are revealing interesting records (Suárez-Morales et al. 2011).

A periodical revision of the progress of the knowledge of the copepod fauna in Costa Rica is a key tool to evaluate their potential diversity in marine and freshwater environments. It is also useful to detect invasive species in different aquatic habitats, which is a worldwide phenomenon representing a serious threat to the aquatic biodiversity (Molnar et al. 2008). The present paper summarizes the information about copepods that has been recorded until now in Costa Rica, a country with two coastlines and with a high habitat diversity.

## Methods

### Surveyed aquatic systems in Costa Rica

**Figure 1.**
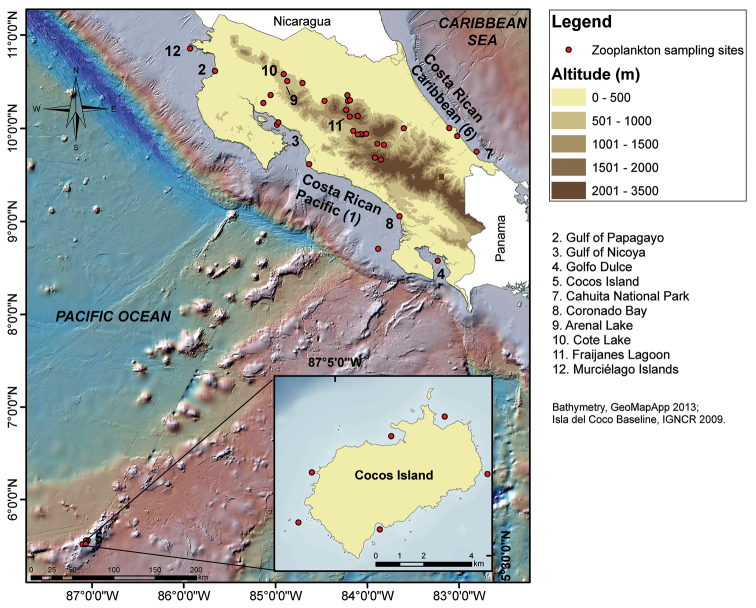
Maps with the sampling sites for marine and freshwater copepods in Costa Rica.

The main coastal and oceanic environments and also the lakes and freshwater sites in which copepods have been surveyed in Costa Rica are presented in Figure 1. These areas and systems were selected because of their physiographic or ecological features: the northern Pacific coast is influenced by the Gulf of Papagayo jet-driven upwelling system (Mc Creary et al. 1989, Chelton et al. 2000). The Gulf of Nicoya, located in the central Pacific Costa Rican coast is one of the largest and well-studied tropical estuaries, with a surface area of 1530 km^2^ (Vargas 1995), and Golfo Dulce to the south is an anoxic fjord-like embayment (Svendsen et al. 2006). The Cocos Island is the only emergent point of an oceanic submarine ridge (Protti et al. 2012), situated 496 km off Cabo Blanco, Pacific coast, with a important marine diversity (Cortés 2012), and since 1997 a UNESCO World Heritage Site (Morales-Ramírez 2008). The Caribbean coast is represented mainly by river deltas dominated by waves and barrier beaches to the north, and sandy beaches alternating with a few formations of coral reef fossils to the south (Denyer and Cárdenas 2000). The most studied freshwater bodies are Lake Arenal, studied since the 1980’s, and smaller lakes like Cote and Fraijanes (Collado et al. 1984a, b, Umaña and Collado 1990).

We reviewed literature records of free-living marine, brackish water, and freshwater copepods found in Costa Rica. Up to thirty seven sites have been sampled for copepods in the country, including marine and freshwater environments (Fig. 1). Marine pelagic copepods were sampled using different gears including WP-2 standard zooplankton nets with mesh sizes of 100, 150, 200, and 500 µm. Vertical tows were performed at depths less than 100 m in Golfo Dulce and Cocos Island (Morales-Ramírez and Nowaczyk 2006, Quesada-Alpízar and Morales Ramírez 2006, Morales-Ramírez 2008). The same kind of tows were used to sample copepods in the Gulf of Papagayo (Bednarski and Morales-Ramírez 2004, Rodríguez-Saénz and Morales-Ramírez 2012), Gulf of Nicoya and Coronado Bay (Morales-Ramírez 1996). In the Caribbean Sea, organisms were collected mostly with horizontal surface tows (Morales-Ramírez and Murillo 1996, Carrillo 2012). For marine benthic copepods, sampling techniques were described by Mielke (1992, 1994a, b, c). This community was also sampled by collecting sediment samples with cores from beaches (Mielke 1992, 1994a, b, c, 1995), but also by a van Veen grab sampler (Sibaja-Cordero 2012). Freshwater copepods were obtained by different devices but mainly by nets used in different habitats including littoral and limnetic areas of the water bodies (see Collado et al. 1984a, b, Suárez-Morales and Reid, unpubl. data).

## Results and discussion

### Historical overview

#### Marine pelagic copepods

The copepod species found in marine systems of Costa Rica are listed in Table 1. The zooplankton of the Pacific coast of Costa Rica has been surveyed more intensely and during a longer period of time than in the Caribbean side. The three sectors (northern, central and southern) of the Pacific coast include interesting systems in which the copepod fauna has been studied. In Culebra Bay, an embayment associated to the Gulf of Papagayo (north Pacific coast), Bednarski (2001) recorded 39 species. Suárez-Morales and Morales-Ramírez (2001) reported the calanoid *Acartia negligens* Dana, 1849 near Murciélago Islands at the northern Pacific coast of Costa Rica and expanded its known regional range. In the same region, a new species (*Cymbasoma concepcionae* Suárez-Morales and Morales-Ramírez 2003) of the order Monstrilloida was described; it was the first record of monstrilloids in Costa Rica. Also in the northern Pacific sector, an oceanic upwelling system known as the Costa Rica Dome has been the subject of many zooplankton studies because of its importance as a fisheries region (Fiedler 2002). A total of 41 copepod species have been recorded in surface waters of this highly productive area (Suárez-Morales and Gasca 1989, Fernández-Alamo and Vicencio 1996).

**Table 1.** List of Copepoda found in marine environments of Costa Rica. Records from published works of the Costa Rican marine copepod fauna. Localities in Costa Rica: 1 – Costa Rican Pacific, 2 – Gulf of Papagayo, 3 – Gulf of Nicoya, 4 – Golfo Dulce, 5 – Cocos Island, 6 – Costa Rican Caribbean, 7 – Cahuita National Park. References: 1. Morales-Ramírez and Suárez-Morales (2009), 2. Carrillo (2012), 3. Sibaja-Cordero (2012), 4. Morales-Ramírez et al. (unpubl. data), 5. Morales-Ramírez and Vargas (1995), 6. Suárez-Morales et al. (2013), 7. Suárez-Morales and Morales-Ramírez (2009), 8. Morales-Ramírez (2001).

Family	Species	Locality	References
Gymnoplea			
Order Calanoida			
Acartiidae	*Acartia clausi* Giesbrecht, 1889	1, 3, 4, 5	1, 4
	*Acartia danae* Giesbrect, 1889	1, 3, 4, 5	1, 4
	*Acartia lilljeborgii* Giesbrecht, 1889	1, 2, 3, 4, 5	1, 4
	*Acartia negligens* Dana, 1849	2	1
Aetideidae	*Aetideopsis rostrata* G.O. Sars, 1905	1	1
	*Aetideus armatus* Boeck, 1872	1	1
	*Aetideus giesbrechti* (Cleve, 1904)	5	4
	*Chiridius* Giesbrecht, 1892	1	1
	*Euchirella brevis* G.O. Sars, 1905	1	1
	*Euchirella rostrata* Claus, 1866	7	1
	*Euaetideus giesbrechti* Sars, 1925	1	1
	*Gaetanus brevispinus* Sars, 1903	1	1
	*Gaetanus miles* Giesbrecht, 1888	1	1
	*Gaetanus minor* Farran, 1905	1	1
	*Gaidius tenuispinus* Sars, 1900	1	1
	*Valdiviella brevicornis* Steuer, 1904	1	1
Arietellidae	*Arietellus* sp. Giesbrecht, 1892	1	1
Augaptilidae	*Augaptilus longicaudatus* Giesbrecht, 1889	1	1
	*Augaptilus megalurus* Giesbrecht, 1889	7	2
	*Haloptilus acutifrons* Giesbrecht, 1892	1	1
	*Haloptilus longicornis* Claus, 1863	1, 6	1
	*Haloptilus mucronatus* Claus, 1863	1	1
	*Haloptilus ornatus* Giesbrecht, 1892	1	1
	*Haloptilus oxycephalus* Giesbrecht, 1889	1	1
Calanidae	*Neocalanus cristatus* (Krøyer, 1848)	1	1
	*Calanus pacificus* Brodsky, 1948	5	4
	*Mesocalanus tenuicornis* (Dana, 1849)	1	1
	*Canthocalanus pauper* (Giesbrecht, 1888)	1, 2, 3, 4, 5	1, 4
	*Cosmocalanus darwini* (Lubbock, 1860)	1, 5	1, 4
	*Nannocalanus minor* Claus, 1863	1, 3, 4, 5	1, 4
	*Neocalanus gracilis* Dana, 1849	1, 2, 5	1, 4
	*Neocalanus robustior* (Giesbrecht, 1888)	1, 5	1, 4
	*Undinula vulgaris* Dana, 1842	1, 2, 3, 4, 5, 6, 7	1, 2, 4
Calocalanidae	*Calocalanus pavo* Dana, 1849	1, 5, 7	1, 2, 4
	*Calocalanus pavoninus* Farran, 1926	1	1
	*Calocalanus styliremis* Giesbrecht, 1888	1, 3, 4	1
	*Calocalanus contractus* Farran, 1926	1	1
	*Ishnocalanus plumulosus* Claus, 1863	1	1
Candaciidae	*Candacia catula* Giesbrecht, 1881	1, 2, 3, 5	1, 4
	*Candacia truncata* Dana, 1846	1, 5	1, 4
	*Candacia pachydactyla* Dana, 1849	1, 5	1, 4
	*Candacia varicans* Giesbrecht, 1892	1	1
Centropagidae	*Centropages abdominalis* (Sato, 1913)	5	4
	*Centropages bradyi* (Wheeler, 1900)	5	4
	*Centropages calaninus* (Dana, 1849)	1, 5	1, 4
	*Centropages furcatus* (Dana, 1849)	1, 5	1, 4
	*Centropages gracilis* (Dana, 1849)	5	4
	*Centropages longicornis* Mori, 1932	1, 5	1, 4
Clausocalanidae	*Clausocalanus arcuicornis* (Dana, 1849)	1, 5	1, 4
	*Clausocalanus furcatus* Brady, 1883	1, 5	1, 2, 4
	*Clausocalanus pergens* Farran, 1926	1, 3, 4	1
Eucalanidae	*Eucalanus attenuatus* Dana, 1849	1, 5	1, 4
	*Eucalanus bungii* Giesbrecht, 1892	1	1
	*Eucalanus crassus* (Giesbrecht, 1888)	7	2
	*Eucalanus elongatus* Dana, 1849	1	1
	*Eucalanus inermis* Griesbrecht, 1892	1	1
	*Eucalanus monachus* Giesbrecht, 1888	6	1
	*Eucalanus mucronatus* Giesbrecht, 1891	1	1
	*Eucalanus pileatus* Giesbrecht, 1888	1	1
	*Eucalanus sewelli* Fleminger, 1973	1, 5	1, 4
	*Eucalanus subcrassus* Giesbrecht, 1888	5, 6	1, 2, 4
	*Eucalanus subtenuis* Giesbrecht, 1888	1, 5	1, 4
	*Rhincalanus cornutus* Dana, 1849	5, 6	1, 4
	*Rhincalanus nasutus* Giesbrecht, 1888	1, 5	1, 4
Euchaetidae	*Euchaeta acuta* Giesbrecht, 1892	1	1
	*Euchaeta plana* Philippi, 1843	5	4
	*Euchaeta barbata* Brady, 1883	1	1
	*Euchaeta indica* Wolfenden, 1905	1, 5	1, 4
	*Euchaeta longicornis* Giesbrecht, 1888	1, 5	1, 4
	*Euchaeta marina* (Prestandrea, 1833)	1, 5	1, 4
	*Euchaeta media* Giesbrecht, 1888	1	1
	*Euchaeta rimana* (Bradford, 1974)	5	4
	*Euchaeta tenuis* Esterly, 1906	1	1
	*Euchaeta wolfendeni* Scott, 1909	1	1
	*Paraeuchaeta hansenii* (With, 1915)	1	1
	*Paraeuchaeta norvegica* (Boeck, 1872)	1	1
	*Paraeuchaeta tonsa* (Giesbrecht, 1895)	1	1
Heterorhabdidae	*Heterorhabdus papilliger* Claus, 1863	1	1
	*Scaphocalanus* sp. G.O. Sars, 1900	1	1
Lucicutiidae	*Lucicutia bicornuta* Wolfenden, 1911	1	1
	*Lucicutia flavicornis* Claus, 1963	1, 5, 6	1, 4
	*Lucicutia gemina* Farran, 1926	1	1
	*Lucicutia grandis* Giesbrecht, 1895	1	1
	*Lucicutia ovalis* Giesbrecht, 1889	1	1
Mecynoceridae	*Mecynocera clausi* Thompson, 1888	1, 5	1, 4
Metridinidae	*Metridia* sp. Boeck, 1864	1	1
	Pleuromamma abdominalis f. edentata Steuer, 1931	1	1
	Pleuromamma abdominalis f. abyssalis Steuer, 1931	1	1
	*Pleuromamma abdominalis abdominalis* Lubbock, 1856	1	1
	*Pleuromamma gracilis* Claus, 1863	1, 5	1, 4
	*Pleuromamma piseki* Farran, 1929	1	1
	*Pleuromamma quadrungulata* Dahl, 1893	1	1
	*Pleuromamma robusta* Dahl, 1893	1	1
	*Pleuromamma scutullata* Brodsky, 1950	1	1
	*Pleuromamma xiphias* (Giesbrecht, 1889)	1	1
Paracalanidae	*Acrocalanus gibber* Giesbrecht, 1888	1, 3, 4, 5	1, 4
	*Acrocalanus gracilis* Giesbrecht, 1888	1, 5	1, 4
	*Acrocalanus longicornis* Giesbrecht, 1888	1	1, 2
	*Paracalanus aculeatus* Giesbrecht 1888	1, 5	1, 2
	*Parvocalanus crassirostris* Dahl, 1894	1	1
	*Paracalanus parvus* Claus, 1863	1	1
Phaennidae	*Cephalophanes* sp. Sars, 1907	1	1
	*Cornucalanus* sp. Wolfenden, 1905	1	1
	*Phaenna spinifera* Claus, 1863	1, 5	1, 4
Pontellidae	*Calanopia americana* F. Dahl, 1894	1, 5, 6	1, 4
	*Labidocera acuta* Dana, 1849	1, 5	1, 4
	*Labidocera aestiva* Wheeler, 1901	1, 5, 7	1, 2, 4
	*Labidocera dentruncata* (Dana, 1849)	5	4
	*Labidocera lubboki* Giesbrecht, 1892	1	1
	*Labidocera scotti* Giesbrecht, 1897	7	1, 2
	*Pontella agassizii* Giesbrecht, 1895	1	1
	*Pontella mimocerami* Fleminger 1957	6	1
	*Pontellina plumata* Dana, 1849	1, 5	1, 4
	*Pontellopsis villosa* Brady, 1883	1	1
	*Pontellopsis yumadae* (Mori, 1937)	5	4
Pseudodiaptomidae	*Pseudodiaptomus acutus* Dahl, 1894	6	1
	*Pseudodiaptomus cristobalensis* Marsh, 1919	1	1
	*Pseudodiaptomus marshi* Wright, 1936	6	1
	*Pseudodiaptomus panamensis* Walter, 1989	3	8
	*Pseudodiaptomus wrigthi* Johnson, 1964	1	1
Scolecithricidae	*Amallothrix gracilis* Sars, 1925	1	1
	*Lophothrix* sp. Giesbrecht, 1895	1	1
	*Scolecithricella dentata* (Giesbrecht, 1892)	1	1
	*Scolecithricella marginata* (Giesbrecht, 1888)	1, 3, 4	1
	*Scolecithricella tenuiserrata* (Giesbrecht, 1892)	1	1
	*Scolecithricella vittata* (Giesbrecht, 1892)	1	1
	*Scolecithricella bradyi* (Giesbrecht, 1888)	1, 5	1
	*Scolecithrix danae* Lubbock, 1856	1, 5, 6	1, 4
	*Scottocalanus* sp. Sars, 1905	1	1
Temoridae	*Eurytemora* Giesbrecht, 1881	1	1
	*Temora discaudata* Giesbrecht, 1889	1, 5	1, 4
	*Temoropia mayumbaensis* Scott, 1894	1, 3, 4, 5	1, 4
	*Temora stylifera* (Dana, 1849)	5, 7	2, 4
	*Temora turbinata* Dana, 1849	1	1, 2
Super Order PODOPLEA			
Order CYCLOPOIDA			
Corycaeidae	*Corycaeus bremhi* Dana, 1849	1	1
	*Corycaeus catus* Dana, 1845	5	4
	*Corycaeus clausi* Dahl F., 1894	7	2
	*Corycaeus crassiusculus* (Dana, 1848)	5	4
	Corycaeus (Agetus) flaccus Giesbrecht, 1891	1, 3, 4	1
	*Corycaeus furcifer* (Claus, 1863)	5	4
	*Corycaeus latus* (Dana, 1848)	5	4
	*Corycaeus limbatus* Brady, 1883	7	2
	Corycaeus (Corycaeus) speciosus Dana, 1849	1, 5, 7	1, 2, 4
	*Corycaeus robustus* (Giesbrecht, 1891)	5	4
	Corycaeus (Onychocorycaeus) ovalis Claus, 1863	1, 5	1, 4
	*Farranula carinata* Giesbrecht, 1891	5	4
	*Farranula gibbula* Giesbrecht, 1981	1, 5	1, 4
	*Farranula gracilis* Dana, 1849	6	1
Oithonidae	*Oithona hebes* Giesbrecht, 1891	6	1
	*Oithona nana* Giesbrecht, 1893	6	2
	*Oithona plumifera* Bair, 1843	1, 5, 6	1, 4
	*Oithona setigera* Claus, 1863	1, 6	1, 2
	*Oithona similis* Claus, 1863	1, 5, 6	1, 4
	*Oithona spinirostris* Claus, 1863	1	1
Oncaeidae	*Conaea gracilis* Dana, 1853	1, 6	1
	*Lubbockia aculeata* Giesbrecht, 1892	1	1
	*Oncaea conifera* Giesbrecht, 1891	1, 5	1, 4
	*Oncaea mediterranea* Claus, 1883	1, 5	1, 4
	*Oncaea ornata* Giesbrecht, 1891	1	1
	*Oncaea venusta* Phillippi, 1843	1, 5, 7	1, 4
	*Pachos punctatum* (Claus, 1863)	5	4
Clausidiidae	*Hemicyclops thalassius* Vervboort & Ramírez, 1966	3	5
Sapphirinidae	*Copilia longistylis* (Mori, 1932)	5	4
	*Copilia mirabilis* Dana, 1852	5, 7	2, 4
	*Copilia quadrata* (Dana, 1852)	5	4
	*Copilia vitrea* Haeckel, 1864	1, 5	1, 4
	*Sapphirina angusta* (Dana, 1849)	5	4
	*Sapphirina darwinii* (Haeckel, 1864)	5	4
	*Sapphirina gastrica* (Giesbrecht, 1891)	5	4
	*Sapphirina metallina* (Dana, 1849)	5	4
	*Sapphirina nigromaculata* Claus, 1863	1, 5	1, 2
	*Sapphirina opalina* Dana, 1849	1, 5	1, 4
	*Sapphirina ovatolanceolata* Dana, 1852	1, 5	1, 4
	*Sapphirina scarlata* Giesbrecht, 1891	1, 5	1, 4
	*Vettoria* sp. Wilson C.B., 1924		
Order MONSTRILLOIDA			
Monstrillidae	*Cymbasoma alvaroi* Suárez-Morales & Carrillo, 2013	7	6
	*Cymbasoma cocoense* Suárez-Morales & Morales-Ramírez, 2009	5	7
	*Cymbasoma concepcionae* Suárez-Morales & Morales-Ramírez, 2003	1	1
	*Monstrilla grandis* Giesbrecht, 1891	7	6
	*Monstrillopsis cahuitae* Suárez-Morales, Carrillo & Morales-Ramírez, 2013	7	6
	*Monstrillopsis chathamensis* Suárez-Morales & Morales-Ramírez, 2009	5	7
Order MORMONILLOIDA			
Mormonillidae	*Mormonilla minor* Giesbrecht, 1891	1	1
	*Mormonilla phasma* Giesbrecht, 1891	1	1
Order HARPACTICOIDA			
Aegisthidae	*Aegistus aculeatus* Giesbrecht, 1891	6	1
Canuellidae	*Microcanuella bisetosa* Mielke, 1994	1	1
Cletodidae	*Cletodes* sp. Brady, 1872	5	3
Clytemnestridae	*Clytemnestra rostrata* Brady, 1883	1, 5	1, 2, 4
	*Clytemnestra scutellata* Dana, 1847	1, 5	1, 2, 4
Ectinosomatidae	*Halectinosoma* sp. Vervoot, 1962	5	3
	Microsetella cf. norvegica (Boeck, 1865)	5	3
	*Microsetella rosea* Dana, 1848	1, 5	1, 4
Diosaccidae	*Balucopsylla triarticulata* Wells & Rao, 1987	1	1
	*Schizopera nicoyana* Mielke, 1995	3	1
	*Schizopera osana* Mielke, 1995	1	1
	*Schizopera* sp. *A* Mielke, 1995	1	1
	*Schizopera* sp. *B* Mielke, 1995	1	1
Harpacticidae	*Zausodes septimus* Lang, 1965	1	1
Laophontidae	*Afrolaophonte schmidti* Mielke, 1997	1	1
	*Klienychocamptoides itoi* Mileke, 1981	1	1
	*Laophontella horrida dentata* Por, 1964	1	1
	*Mexicolaophonte arganoi* Cottarelli, 1977	1	1
Leptastacidae	Leptastacidae undet.	5	3
Longipediidae	*Longipedia helgolandica* Klie, 1949	6	1
Miraciidae	*Amonardia* sp. Lang, 1944	5	3
	Amphiascopsis cf. cinctus (Claus, 1866)	5	3
	*Macrosetella gracilis* Dana, 1852	1, 5	1, 2, 4
	*Robertgurneya* sp. Lang, 1944	5	3
	*Typhlamphiascus* sp. Lang, 1944	5	3
Orthopsyllidae	*Orthopsyllus linearis curvaspina* Claus, 1886	5	3
Paramesochridae	Paramesochridae indet.	5	3
Peltidiidae	*Peltidium nichollsi* Geddes, 1968	6	2
Phyllopodidae	*Phyllopodopsyllus ancylus* Mielke, 1992	1	1
	*Phyllopodopsyllus carinatus* Mielke, 1992	1	1
	*Phyllopodopsyllus gertrudi costaricensis* Kunz, 1984	1	1
	*Phyllopodopsyllus setouchiensis* Kitazima, 1981	1	1
Euterpinidae	*Euterpina acutifrons* Dana, 1852	1, 5, 6	1
Thalestridae	*Amenophia* sp. Boeck, 1865	5	2
Tetragonicipitidae	*Oniscopsis robinsoni* Chappuis & Delamare, 1956	6	1

In the Gulf of Nicoya, an estuarine system on the central Pacific coast of Costa Rica, Morales-Ramírez and Vargas (1995) reported 12 copepod species dwelling at the inner sector of the gulf. They determined *Acartia lilljeborgii* and members of the family Pseudodiaptomidae as the most abundant taxa. Further studies in the Gulf of Nicoya raised the total number of species to 32 (Morales-Ramírez 1996).

Zooplankton studies in the southern Pacific area of Costa Rica have focused on two locations. The first one is Coronado Bay, which is part of the Térraba-Sierpe mangrove system. Its copepod fauna comprised 13 species of a few families, mainly Clausocalanidae, Calanidae and Paracalanidae (Morales-Ramírez 1996). The second location is Golfo Dulce, where Morales-Ramírez (1996) recorded 38 species. The genera *Corycaeus*, *Clausocalanus*, *Oncaea* and *Oithona* were the most common in this area. This gulf harbors 21.5% of the marine diversity recorded from the Costa Rican Pacific coast (Morales-Ramírez 2011).

In the Caribbean coast of Costa Rica, studies on copepods are scarcer and more recent. In the Cahuita National Park, Morales-Ramírez (2001) collected 22 species, with *Acartia lilljeborgii* and species of the family Pontellidae as the dominant forms. At that time, 18 of those species were reported as new records for the Caribbean coast of Costa Rican. Further studies revealed 13 additional records (Carrillo 2012) and two new species of monstrilloid copepods: *Monstrillopsis cahuitae* Suárez-Morales, Carrillo & Morales-Ramírez, 2013 and *Cymbasoma alvaroi* Suárez-Morales & Carrillo, 2013 (Suárez-Morales et al. 2013).

**Figure 2.**
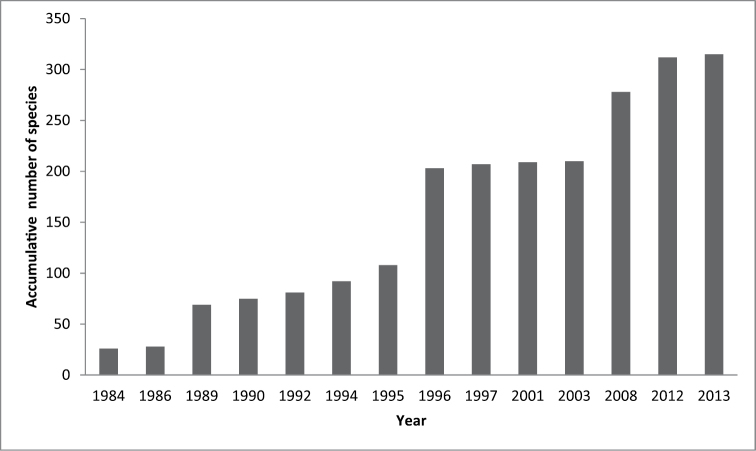
Accumulative number of species of marine and freshwater copepods recorded per year in Costa Rica. Data from the revision of the published literature.

The zooplankton sampling efforts in Costa Rica have been carried out since the the 1980´s. Figure 2 shows the accumulative number of species progressively found by national investigators. There seems to be major contributions to a sustained increase since 1984, nevertheless, the period between 1996 and 2003 added few or none new records; subsequently, with the beginning of studies around Cocos Island National Park increased the number of records. The first studies that included some analysis of copepods as a group (not on a species level) were from the coastal area around the Cahuita National Park, a Caribbean reef system (Morales 1987), and Caño Island at the Pacific coast (Guzmán and Obando 1988). Considering these early surveys, it is concluded that the current knowledge of the copepod fauna comprises more than two decades (Fig. 2). In 2009, the list of species recorded in Costa Rica included 209 species of marine copepods: 185 were planktonic and 24 benthic, 165 in the Pacific coast and 44 in the Caribbean waters. These numbers excluded species from Cocos Island (Morales-Ramírez and Suárez-Morales 2009).

Periodical sampling of the zooplankton of Cocos Island started in 2007, with a 6-station sampling plan around the island and in coral reefs patches. These samples first yielded 68 species of copepods (Morales-Ramírez 2008), a list that now includes 95 species (Table 1), and 14 new records of planktonic copepods, eight benthic forms (Morales-Ramírez et al. unpubl. data), and two new monstrilloids species: *Cymbasoma cocoense* Suárez-Morales & Morales-Ramírez, 2009 and *Monstrillopsis chathamensis* Suárez-Morales & Morales-Ramírez, 2009; the last case represented the first record of the genus *Monstrillopsis* for Costa Rican waters (Suárez-Morales and Morales-Ramírez 2009). The species composition of the copepod fauna from Cocos Island and adjacent oceanic waters has been summarized by Morales-Ramírez (2008). The two most diverse families are Corycaeidae and Sapphirinidae (unpubl. data).

#### Marine benthic copepods

The number of free-living copepod taxa that have been described from material obtained in Costa Rica is summarized in Table 2. Taxonomic surveys of the benthic copepod fauna are scarce. Mielke (1992) analyzed beach sediments from locations along both coasts of Costa Rica: along the Caribbean coast, samples were obtained at three locations (Portete, Cahuita and Manzanillo beaches), whereas at the Pacific coast, samples were obtained from 13 sites. Six copepod taxa belonging to the family Tetragonicipitidae were found, including two new species (*Phyllopodopsyllus ancylus* Mielke, 1992 and *Phyllopodopsyllus carinatus* Mielke, 1992) and two new subspecies (*Laophontella horrida dentata* Mielke, 1992 and *Phyllopodopsyllus gertrudi costaricensis* Mielke, 1992). The monotypic genus *Microcanuella* and its species *Microcanuella bisetosa* Mielke, 1994 were described from sandy beaches of the Gulf of Nicoya. Mielke (1995) also found four species of *Schizopera* Sars G.O., 1905 and described *Schizopera osana* Mielke, 1995.

**Table 2.** New species described from type material collected in Costa Rica. References. 1. Mielke (1994a), 2. Mielke (1992), 3. Mielke (1994c), 4. Mielke (1995), 5. Suárez-Morales and Morales-Ramírez (2003), 6. Suárez-Morales and Morales-Ramírez (2009), 7. Suárez-Morales et al. (2013), 8. Suárez-Morales and Gasca (2012).

Order	Species	Location	Environment	References
Harpacticoida	*Karllangia obscura* Mielke, 1994	Manzanillo, Caribbean coast	Mud flat	1
	*Karllangia pulchra* Mielke, 1994	Manzanillo, Caribbean coast	Mud flat	1
	*Phillopodopsyllus carinatus* Mielke, 1992	Manzanillo, Caribbean coast	Beach slope	2
	*Phillopodopsyllus gertrudi costaricensis* Mielke, 1992	Manzanillo, Caribbean coast	Beach slope and mangrove	2
	*Phyllopodopsyllus ancylus* Mielke, 1992	Punta Morales, Pacific coast	Mud flat	2
	*Orthopsyllus linearis curvaspina* (Claus, 1886)	Pacific coast	Sandy beach	3
	*Microcanuella bisetosa* Mielke, 1994	Gulf of Nicoya, Pacific coast	Sandy beach	3
	*Schizopera nicoyana* Mielke, 1995	Gulf of Nicoya, Pacific coast	Sandy beach	4
	*Schizopera osana* Mielke, 1995	Gulf of Nicoya, Pacific coast	Sandy beach	4
	*Laophontella horrida dentata* (Por, 1964)	Pacific coast	Sandy beach	2
Monstrilloida	*Cymbasoma concepcionae* Suárez-Morales & Morales-Ramírez, 2003	Bahía Culebra, Pacific coast	pelagic	5
	*Cymbasoma cocoense* Suárez-Morales & Morales-Ramírez, 2009	Cocos Island, Pacific Ocean	pelagic	6
	*Monstrillopsis chathamensis* Suárez-Morales & Morales-Ramírez, 2009	Cocos Island, Pacific coast	pelagic	6
	*Monstrillopsis cahuitae* Suárez-Morales, Carrillo & Morales-Ramírez, 2013	Cahuita National Park, Caribbean coast	pelagic	7
	*Cymbasoma alvaroi* Suárez-Morales, Carrillo & Morales-Ramírez, 2013	Cahuita National Park, Caribbean coast	pelagic	7
Siphonostomatoida	*Lepeophtheirus alvaroi* Suárez-Morales & Gasca, 2012	Cocos Island, Pacific Ocean	water column	8

At Cocos Island, Sibaja-Cordero (2012) analyzed subtidal sediment samples and identified seven families, nine genera and three species of harpacticoid copepods. The family Miraciidae included four genera, one species and an undetermined taxon. This material is expected to reveal many additional species records of the Harpacticoida.

#### Freshwater copepods

The freshwater zooplankton of Costa Rica has been studied since the 1980’s, but these surveys have been intermittent (Collado et al. 1984a, b, Dussart and Fernando 1986, Reid 1990a, Umaña and Collado 1990, Haberyan et al. 1995). Since then, a total of 25 species of copepods have been recorded (Table 3). Cyclopid copepods are the most diverse and abundant group in inland waters of Costa Rica as found in other Neotropical areas (Suárez-Morales et al. 2000, Silva 2008). Diaptomid copepods, the most representative group of the order Calanoida in freshwater environments, show a low diversity in Costa Rica, a feature which is also shared with Central America (Suárez-Morales 2003, Suárez-Morales et al. 2005). Their diversity could be underestimated in Costa Rica; records of presumedly cosmopolitan species like *Eucyclops agilis*, *Eucyclops serrulatus* could include undescribed species (Alekseev et al. 2006, Gutiérrez-Aguirre et al. 2013). The Costa Rican *Eucyclops bondi* probably represents an undescribed species (Mercado-Salas and Suárez-Morales 2014).

**Table 3.** Species of freshwater copepods recorded in Costa Rica (* new range extension includes Costa Rica, ** only known from Costa Rica). References: 1. Collado et al. (1984a), 2. Collado et al. (1984b), 3. Dussat and Fernando (1986), 4. Reid (1990a), 5. Reid (1990b), 6. Gavlas (2012), 7. Suárez-Morales et al. (2013).

Family	Species	References
Gymnoplea		
Order Calanoida		
Diaptomidae	*Arctodiaptomus dorsalis* (Marsh, 1907)	2
	*Diaptomus* sp. Westwood, 1836	1
	*Prionodiaptomus colombiensis* (Thiébaud, 1912)*	6
Super Order PODOPLEA		
Order CYCLOPOIDA		
Cyclopidae	*Ectocyclops pharelatus* (Koch, 1838)	1
	*Eucyclops agilis* (Koch, 1838)	1
	*Eucyclops bondi* Kiefer, 1934	2
	*Eucyclops leptacanthus* Kiefer, 1956	2
	*Eucyclops serrulatus* (Fischer, 1851)	1
	*Halicyclops exiguus* Kiefer, 1934	1
	*Macroyclops albidus* (Jurine, 1820)	1
	*Mesocyclops brasilianus* Kiefer, 1933	1
	*Mesocyclops leuckarti* (Claus, 1857)	1
	*Mesocyclops thermocyclopoides* Harada, 1931	2,7
	*Microcyclops ceibaensis* (Marsh, 1919)	2
	*Microcyclops dubitabilis* Kiefer, 1934	2
	*Microcyclops varicans* (G.O Sars, 1863)	1
	*Paracyclops fimbriatus* (Fischer, 1853)	2
	*Thermocyclops crassus* (Fischer, 1853)	2
	*Thermocyclops decipiens* (Kiefer, 1929)	2
	*Thermocyclops inversus* (Kiefer, 1936)	2
	*Thermocyclops tenuis* (Marsh, 1910)	2
	*Tropocyclops prasinus* (Fishcer, 1860)	2
	*Tropocyclops pseudoparvus* Dussart & Fernando, 1986**	3
Order HARPACTICOIDA		
Canthocamptidae	*Attheyella fuhrmanni* (Thiébaud, 1912)	4
	*Canthocamptus striblingi* (Reid, 1990) **	5

#### General diversity

Studies focusing on the taxonomic composition of the free-living copepod fauna have been carried out in selected locations involving coastal, oceanic and large continental aquatic systems (i.e., lakes as: Arenal, Cote, Bonilla, Fraijanes, and Cerro Chato). Currently, there are 281 species of copepods recorded in Costa Rica, representing 6 orders and 50 families. The order with the highest number of species is Calanoida (147 spp.), followed by Cyclopoida (73) and Harpacticoida (36). There are no records of species of the orders Gelyelloida, Platycopioida and Misophrioida.

According to our results, copepods are the most studied marine group of microcrustaceans in Costa Rica, representing almost 44% of the 473 marine species reported in the country until 2009 (Wehrtmann and Cortés 2009); this figure increases to 52.6% when studies around Cocos Island and Caribbean Sea as well new records from other areas are considered.

## Remarks

### Marine copepods

Being situated in a fully tropical area with the influence of both the Atlantic and Pacific oceans, the marine copepod fauna of Costa Rica is expected to be highly diverse. The analysis of the diversity is also an important tool to reveal changes and patterns of the pelagic communities surveyed. In these coastal systems, particularly along the Pacific coast, the diversity of copepods has detectable variations as a response to local oceanographic conditions. Also, typical upwelling species have been recorded in Culebra Bay, Gulf of Papagayo, with major seasonal changes in dominance as a response to upwelling conditions (Bednarski and Morales-Ramírez 2004). The Gulf of Nicoya is known to show an assemblage of typical estuarine species, where small calanoids dominate year-round (Brugnoli et al. 2004). The Golfo Dulce represents a mixed environment in which oceanic and coastal copepod species coexist, a condition that is intensified during El Niño events (Quesada-Alpízar and Morales-Ramírez 2006). These and other Costa Rican systems should be surveyed during different seasons and hydrographic conditions in order to develop a complete overview of their copepod diversity. Also, increased efforts should be carried out to explore the diversity of benthic copepods, mainly of harpacticoids, a group whose diversity in marine and freshwater environments is still poorly known in Costa Rica.

### Freshwater copepods

Currently, 25% of Central American records of freshwater species are from Costa Rica. Together with that of the other areas of Central America (CA), its inland copepod fauna represents an interesting assemblage, because CA is a recent biogeographic passage of copepods between North and South America (Suárez-Morales 2003, Suárez-Morales et al. 2005). Also, there are records of introduced species of copepods in Costa Rica (Collado et al. 1984a, b, Suárez-Morales et al. 2011); their advancement in the country should be followed based on an expanded sampling program. Additional efforts are required to reveal the copepod diversity of Costa Rican freshwater systems. New inland aquatic habitats should be sampled, like ephemeral pools, subterranean waters, caves, methane seeps, and even semi-terrestrial habitats, often harboring a rich copepod fauna (Reid 1986).

A consistent plan to develop more human resources formed in the taxonomy and systematics of these microcrustaceans is required as a basic strategy to increase the knowledge of this biodiversity. Thereby we could increase the knowledge of our biological diversity and thus allow the development of improved conservation strategies (Mercado-Salas et al. 2013) and prevent or mitigate some of the problems related to the loss of biodiversity (Molnar et al. 2008).
